# Integration of Retinal and Extraretinal Information across Eye Movements

**DOI:** 10.1371/journal.pone.0116810

**Published:** 2015-01-20

**Authors:** Florian Ostendorf, Raymond J. Dolan

**Affiliations:** 1 Berlin School of Mind and Brain, Humboldt-Universität zu Berlin, Berlin, Germany; 2 Dept. of Neurology, Charité—Universitätsmedizin Berlin, Berlin, Germany; 3 Wellcome Trust Centre for Neuroimaging, University College London, London, United Kingdom; 4 Max Planck UCL Centre for Computational Psychiatry and Ageing Research, Russell Square House, London, United Kingdom; VU University Amsterdam, NETHERLANDS

## Abstract

Visual perception is burdened with a highly discontinuous input stream arising from saccadic eye movements. For successful integration into a coherent representation, the visuomotor system needs to deal with these self-induced perceptual changes and distinguish them from external motion. Forward models are one way to solve this problem where the brain uses internal monitoring signals associated with oculomotor commands to predict the visual consequences of corresponding eye movements during active exploration. Visual scenes typically contain a rich structure of spatial relational information, providing additional cues that may help disambiguate self-induced from external changes of perceptual input. We reasoned that a weighted integration of these two inherently noisy sources of information should lead to better perceptual estimates. Volunteer subjects performed a simple perceptual decision on the apparent displacement of a visual target, jumping unpredictably in sync with a saccadic eye movement. In a critical test condition, the target was presented together with a flanker object, where perceptual decisions could take into account the spatial distance between target and flanker object. Here, precision was better compared to control conditions in which target displacements could only be estimated from either extraretinal or visual relational information alone. Our findings suggest that under natural conditions, integration of visual space across eye movements is based upon close to optimal integration of both retinal and extraretinal pieces of information.

## Introduction

Saccades are fast ballistic eye movements that help us to acquire high-quality information about relevant aspects of a visual scene. Since every saccadic eye movement displaces retinal representations, saccades also represent a potentially disturbing event for the maintenance of stable representations of the visual world. Different mechanisms may help to disambiguate self-induced displacements due to our eye movements from external motion in the outside world. One mechanism is thought to involve an internal monitoring of eye position. Experimental evidence [1–3] suggests a predominant role of oculomotor “outflow” [4] rather than proprioceptive “inflow” [5] signals in this internal monitoring across single saccadic eye movements by using an efference copy [6] or corollary discharge (CD) [7] of the oculomotor command (but see other findings, suggesting a sizeable influence of proprioceptive inflow for the perception of straight ahead in the dark [8] and the matching of visual space across multiple saccade sequences [9, 10]).

Perfect compensation of self-induced displacements by CD requires a highly reliable reproduction of saccade dynamics. Probing the quality of CD-dependent visual stability across eye movements in psychophysical set-ups has yielded equivocal findings. Specifically, detection of visual displacements is degraded if stimulus jumps occur in sync with a saccade, a phenomenon termed saccadic suppression of displacement (SSD) [11–14]. Moreover, briefly flashed stimuli undergo gross perceptual mislocalization around saccade onset [15–17]. These findings suggest limited accuracy and precision of internal monitoring signals, both in the temporal and spatial domain. On the other hand, perisaccadic mislocalization may primarily be of visual origin [18, 19] and localization of perisaccadic flashes can indeed be veridical when a directed motor response is required with visual references extinguished [20, 21]. Furthermore, the spatial matching of stimuli across saccades dramatically improves with small changes in experimental design: Blanking the saccade target for a short time restores sensitivity to its displacement [12, 22]. Precise and accurate extraretinal information on eye movement dynamics is thus in principle available and can—at least under certain circumstances—be used to deal efficiently with self-induced displacements of retinal representations.

Laboratory studies that address CD-dependent maintenance of visual stability classically employ sparse visual displays, devoid of contextual information [1, 2, 12, 20]. By contrast, everyday life exposes the visuomotor system to visual scenes that are typically populated with a manifold of different objects. We reasoned that spatial relational information between objects should constitute a complementary source of information. Here we take our lead from James Gibson’s influential formulation of the *optic array* [23], where experimental findings suggest that matching of visual space across eye movements is strongly driven by visual context information, whenever this source of evidence is available [24, 25]. Specifically, these studies suggest a privileged processing of visual information around the saccade target: A rough match of target location and identity across the saccade may suffice to realign surrounding scene context even to larger intra-saccadic target jumps and preserve the subjective impression of visual stability. When the target object is temporarily blanked, continuously available flanker objects can take over this anchoring function, biasing detection of target displacements [25]. This is reminiscent of visual capture, i.e. a strong dominance of visual information over other sources of information in multisensory integration [26].

However, from a normative perspective, perceptual decisions should take into account both extraretinal information and visual relational cues: Integrating both pieces of evidence according to their relative reliability should lead to more precise and less biased estimates [27]. Close to optimal integration has been demonstrated for a variety of sensory channels in the past [28–31] and a recent study suggested a similar mechanism for the integration of visual, proprioceptive and CD information for saccade sequences in the dark [10]. We reasoned that the evaluation of perceptual stability across eye movements might involve the efficient integration of extraretinal and visual relational information. We asked healthy human observers to report the apparent jump of a saccade target that was temporarily switched off during saccades and reappeared with an unpredictable displacement afterwards. Correct decisions about jump direction require precise and accurate knowledge of eye movement metrics [12], presumably mediated by CD [32–35]. Results demonstrate that perceptual decisions in this task benefit from additional, visual relational information, offered by a non-target flanker object. Perceptual bias and precision closely corresponded to predictions from a simple model of optimal cue integration that assumes a weighted averaging of both extraretinal and visual relational information, depending on their respective reliability.

## Materials and Methods

### Observers

Four healthy human subjects (one male, three naïve; mean age, 28 years; age range, 22–39 years) participated in this study. All subjects had normal vision and gave written informed consent before participation. The study was approved by the local ethics committee (Charite´—Universitätsmedizin Berlin, Germany, EA1/212/11) and conducted in conformity with the Declaration of Helsinki.

### Experimental set-up

Subjects sat at a viewing distance of 57 cm in front of a 22-inch CRT-monitor (Eizo Flexscan F931; resolution 1024 × 768 pixels; refresh rate, 140 Hz) with their heads stabilized by a chin- and headrest. Eye movements were monitored for the right eye with high-speed video-oculography (Eyelink 2K, SR Research) at a 1000-Hz sampling rate.

### Task and procedure

Experiments were carried out in an otherwise darkened room. Subjects completed the experiment in multiple test sessions on separate days. Three different task conditions (JUMP, VISUAL and COMBI) were tested separately in subsequent sessions and instructions for an upcoming condition were given before a corresponding session. Half of the trials for VISUAL and then JUMP condition were conducted before COMBI condition on first day and the rest of trials in reverse order on a second day. We aimed at collecting 12, 6 and 12 blocks (36 trials per block) for VISUAL, JUMP and COMBI condition, respectively. Stimuli were presented on a homogenous gray background (20.1 Cd/m^2^). A relatively high background luminance was chosen to exclude any spurious effects of phosphor persistence. Previous work demonstrated that visible screen borders should not significantly influence localization with our stimulus configuration [25]. Visual presentation was realized by using Matlab (The Mathworks, Natick, MA) with the Psychophysics and Eyelink Toolbox extensions [36–38].

**Figure 1:**
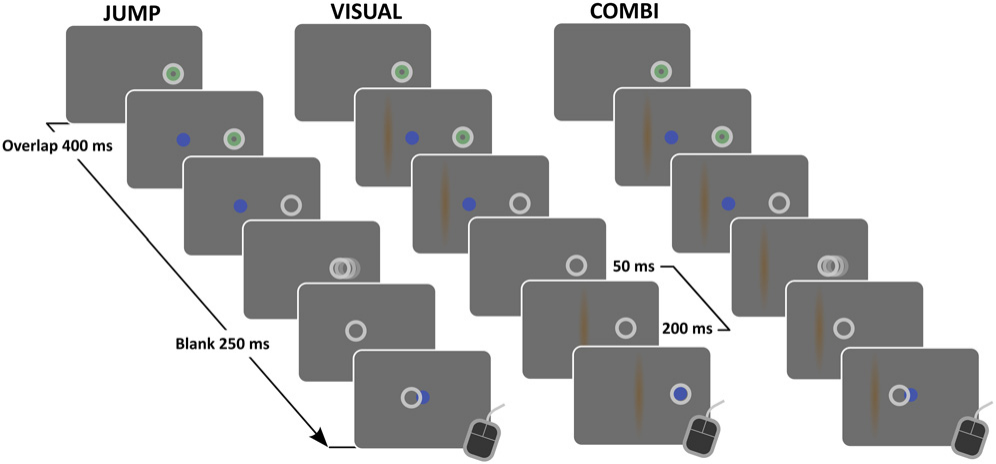
Cartoon of experimental conditions. In JUMP condition (left), after an unpredictable foreperiod of stable fixation (1200–1600 ms; eye position, white circles) on an initial fixation dot (green), a second dot (blue) appeared 10 deg to left of fixation. After 400 ms stimulus overlap, the fixation dot was turned off, serving as a signal to perform a saccadic eye movement to the second dot. Contingent on saccade onset, the target dot was switched off and reappeared 250 ms later with variable and unpredictable horizontal offset. Subjects performed a perceptual judgment on the apparent jump direction by means of a button press. In the VISUAL condition (middle), the trial started similar to JUMP condition, including the stimulus overlap, but with the exception of a second object (orange vertical bar) that appeared together with the target dot. Subjects kept initial fixation when the fixation cue disappeared and target and flanker object remained on screen for another 200 ms (mimicking saccadic reaction time). Then all cues disappeared for 50 ms (mimicking the visual transient induced by the saccade in JUMP and COMBI condition). Then, first the flanker and then the target reappeared (onset asynchrony, 200 ms, mimicking remaining BLANK duration after saccade in COMBI condition), with target position now coinciding with fixation and flanker position coinciding with previous relative position to target dot plus some variable and unpredictable offset. Here, subjects indicated the apparent relative displacement of the flanker object with respect to the target position. In COMBI condition (right), the task was identical to JUMP condition plus the flanker object of the VISUAL task. This flanker object remained on screen at same position throughout the trial.

In all experimental tasks, a red dot (diameter, 0.7 deg) was presented at 6 deg right of screen center (coinciding with body midline of subject). When fixation of this dot was detected, the dot turned into green color. After a variable foreperiod (drawn from uniform distribution, 1200–1600 ms), a blue dot (diameter, 0.7 deg, luminance, 5 Cd/m^2^) was presented at 4 deg left of screen center (JUMP task, Fig. 1, *left*). After 400 ms of stimulus overlap, the initial fixation cue was switched off, serving as a go-signal to execute a saccadic eye movement to the target. Contingent on the eye movement, the target was temporarily switched off and reappeared 250 ms later at an unpredictable position. Gaze-contingent stimulus offsets were triggered by on-line detection of saccade onsets. Specifically, we computed the sum of absolute differences between current eye position sample and the running average of the last 6 samples for horizontal and vertical eye position, compared against a threshold value of 0.3 deg. Pilot testing confirmed that this position-based algorithm (as originally proposed by the eyetracker’s manufacturer) provided for a sensitive but robust detection of saccade onsets for our set-up. We confirmed by offline analysis that target extinction took place within the first half of saccades [mean delay (±1 S.D.) between saccade start and display change with the latter captured by timestamp of corresponding screen refresh command, 13.2 (±0.33) ms versus mean saccade duration, 44.1 (±1.9) ms]. We note that this delay does not account for small additional delays between refresh command and actual screen refresh (i.e., input lag). These additional time delays should however be small and we explicitly checked for missed frames with our experimental code. After saccade completion and subsequent reappearance of the displaced target, subjects performed a perceptual judgment on the apparent jump direction (by pressing left or right mouse button with index or middle finger of right hand, respectively). Response time was limited to 5 s and the target disappeared when a button press was recorded or maximum response time had elapsed. The screen was then blanked for 1400 ms and a next trial started. Target displacement for a given trial was adapted by three independent, randomly interleaved staircases with a constant step size of 1 deg. Specifically, when the subject indicated a target displacement to the left for a given displacement level, the next displacement level for a given staircase would be shifted by 1 deg to the right, i.e., staircases followed a one-up, one-down logic. Staircases started at a displacement level of 1.33 deg right- and leftward and 0 deg (no displacement) with respect to initial target position. Interleaved displacement levels for the three staircases enabled sampling the point of subjective target constancy with a resolution of .33 deg while collecting a sufficient number of trials at higher confidence levels.

In the COMBI task, a flanker object (thin vertical orange bar, minimum luminance, 14.7 Cd/m^2^, two-dimensional ellipsoid gaussian, standard deviation 0.175 deg X 4 deg) was presented together with the saccade target at 1.2, 2.8 or 4.4 deg to the left of target position (Fig. 1, *right*). This flanker object stayed on screen until trial end. The task was otherwise identical to the JUMP task. In the VISUAL task (Fig. 1, *middle*), target and flanker object were first presented in the periphery together with the fixation dot for 400 ms of stimulus overlap, identical to the COMBI task. However, subjects were instructed to keep fixating on the initial fixation position when the fixation dot disappeared and only the peripheral target-flanker pair remained on for another 200 ms (mimicking saccadic reaction time), followed by a brief blank period (duration, 50 ms, mimicking the visual transient induced by the saccade). Then, first the flanker and then the target object reappeared, both objects now displaced toward subject’s fixation (stimulus onset asynchrony, 200 ms, reproducing the blank period in JUMP and COMBI task). Specifically, the target was presented at fixation and the flanker at the initial target-flanker distance plus an unpredictable displacement. The VISUAL task thus mimicked the retinal stimulation and timing of the COMBI task without the intervening saccade (and any associated idiosyncratic saccade targeting errors). Subjects were instructed to judge the relative displacement of the flanker with respect to the target, as pilot testing showed this judgement to be easier to perform with the target location coinciding with fixation than judging the displacement of the target relative to the flanker.

### Data analysis

Eye movement data were low-pass filtered (zero-phase second-order butterworth filter; cut-off frequency, 50 Hz), visualized and analyzed in Matlab by using self-written routines. Saccade onset and offset were determined by using a velocity criterion (threshold, 25 deg/s; minimum duration, 10 ms). Saccade start and end positions were determined as the preceding and following fixation periods (defined by a dispersion criterion; 0.5 deg maximum dispersion for minimum duration of 40 ms). Trials with anticipatory saccades [eye position leaving an imaginary circular window around fixation (radius, 1 deg)] large saccade targeting errors (absolute error > 6 deg) or blinks before or during first saccade execution were excluded from further analysis in the JUMP and COMBI task (mean exclusion rate, 4%). Trials with fixation breaks during peripheral target presentation or during the following brief blanking period in the VISUAL condition were aborted during the experiment and repeated later in the experimental block. During offline analysis, we applied the saccade detection criteria stated above (eye velocity of at least 25 deg/s for at least 10 ms) for further exclusion of trials with small saccades that escaped the online detection algorithm during the critical trial periods (i.e., overlap phase and subsequent 200 ms; average exclusion rate, 2.9%).

Cumulative gaussians were fitted to perceptual response data in Matlab by using *psignifit*, a toolbox that implements the maximum-likelihood method described by [39]. We included a small lapse rate parameter (λ < 0.05) that accounts for stimulus-independent errors (lapses or mistakes) of the subjects. Displacement thresholds and the point of subjective stationarity (PSS) were described by the estimated standard deviation and mean of the fitted psychometric function. To simulate performance in the COMBI condition as integration of internal monitoring and afferent relational cues, we used predictions from a simple cue integration model (maximum likelihood estimation, MLE) that corresponds to optimal integration under certain assumptions (independent gaussian noises for both estimates, a uniform, noninformative prior). We used perceptual performance in the VISUAL and JUMP task as a proxy to the retinal (*RET*) and extraretinal (*EXTRA*) estimates of target jumps that are available to the observer in the COMBI task. Following MLE, an optimal observer should integrate these two single-cue estimates in a way that the perceptual bias (PSS) in the COMBI task corresponds to,

$$PSS_{COMBI}=w_{RET}\times PSS_{RET}+\left(1-w_{RET}\right)\times PSS_{EXTRA}$$ (1)

with *w_RET_* denoting the relative weight for the afferent cue, proportional to its inverse relative variance:

$$w_{RET}=\frac{\frac{1}{\sigma_{RET}^{2}}}{\frac{1}{\sigma_{RET}^{2}}+\frac{1}{\sigma_{EXTRA}^{2}}}$$ (2)

A further and even stronger prediction of optimal integration is that the variance of the combined estimate will always be less than either individual estimate [27]. From single-cue thresholds in the JUMP and VISUAL condition, we therefore computed thresholds for an optimal observer in the COMBI task:

$$\sigma_{COMBI}^{2}=\frac{\sigma_{RET}^{2}\times \sigma_{EXTRA}^{2}}{\sigma_{RET}^{2}+\sigma_{EXTRA}^{2}}$$ (3)

We then compared predicted thresholds with the thresholds observed in the combined condition.

## Results

**Figure 2:**
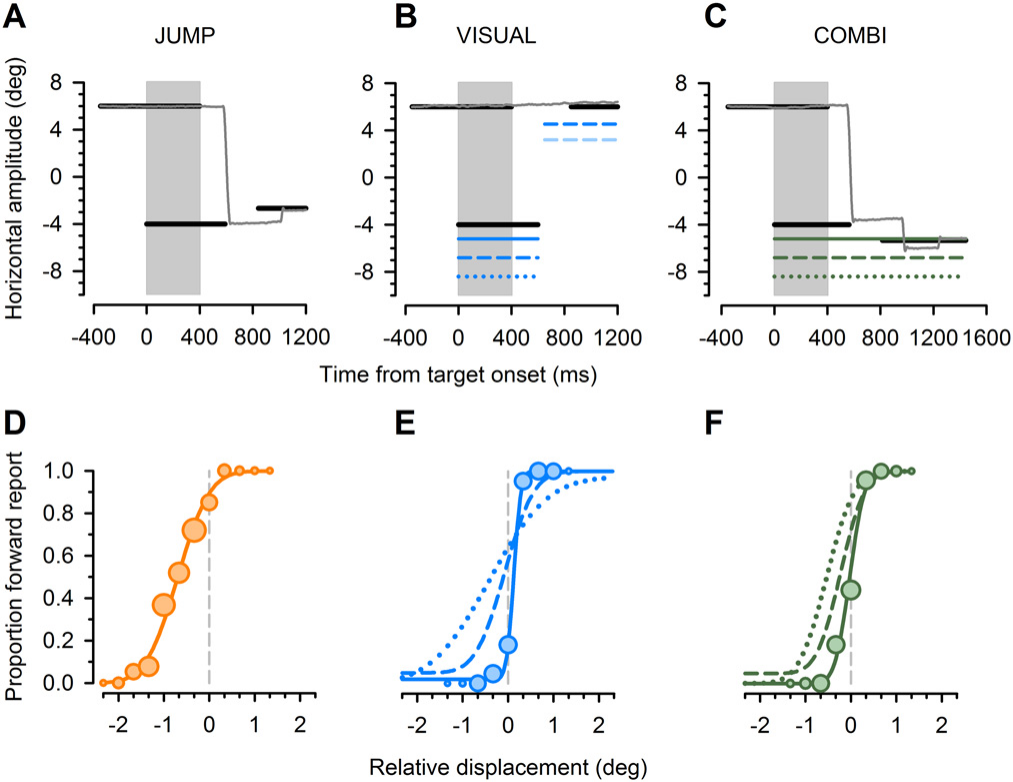
Task schematic and exemplary results. ***A***, Trials started with a fixation cue and a target 10 degree to the left (black lines). After 400 ms of stimulus overlap (shaded area), the fixation was switched off, serving as go signal to perform a saccadic eye movement to the target (exemplary eye trace in horizontal plane, gray line). Saccade onset triggered a target blank for 250 ms (JUMP task), after which the target reappeared with unpredictable offset. Subjects indicated apparent jump direction. ***B***, In the VISUAL task, subjects kept initial fixation while the peripheral target and an additional flanker object (vertical bar) was presented at one of three distances [at 1.2, 2.8 (this trial) and 4.4 deg left of target; blue continuous, dashed and dotted lines, respectively]. The fixation dot then disappeared and 200 ms later, the target-flanker pair was briefly switched off and reappeared in the center field of view, mimicking the visual consequences of an intervening saccade to the target. Critically, the target-flanker distance was now changed (shown only for actual flanker position with fainter color corresponding to the unchanged relative position) and subjects reported the apparent change in relative position of the flanker with respect to the target. ***C***, In the COMBI task, stimuli and instructions were identical to the JUMP task, apart from the additional flanker object that was presented together with the target at one of three target-flanker distances [at 1.2, 2.8 (this trial) and 4.4 deg left of target; green continuous, dashed and dotted lines, respectively]. ***D-F***, Psychometric functions of one naïve subject. Proportion of trials in which subject reported an apparent target jump in saccade direction (forward), plotted against relative displacement levels. Negative values refer to target displacements against saccade direction. Circle sizes represent the number of trials for a given target jump. Cumulative Gaussians were fitted to perceptual response data, separately for different conditions [JUMP (***D***), VISUAL (***E***), COMBI (***F***)] and the three different target-flanker distances in VISUAL and COMBI condition (1.2 deg, continuous lines; 2.8 deg, dashed lines; 4.4 deg, dotted lines). Raw data for medium and large distance are not shown for the sake of clarity.

**Figure 3:**
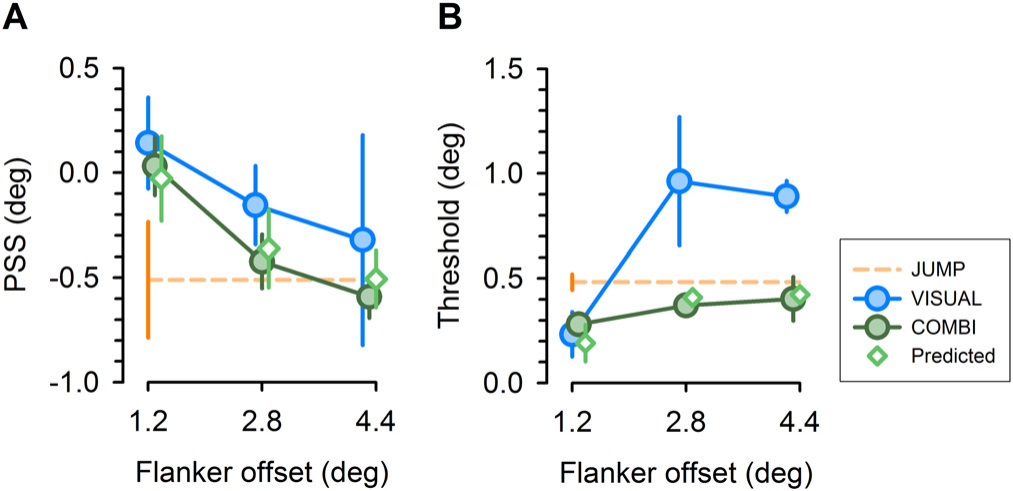
Group average perceptual performance, shown for PSS (left panel) and threshold estimates (right panel) of fitted psychometric functions, respectively. Diamonds represent predictions for COMBI condition, as expected from average estimates in single cue conditions. Error bars represent within-subject standard errors of mean [50]. Data points for different conditions are shown with small horizontal offsets to increase visibility.

**Figure 4:**
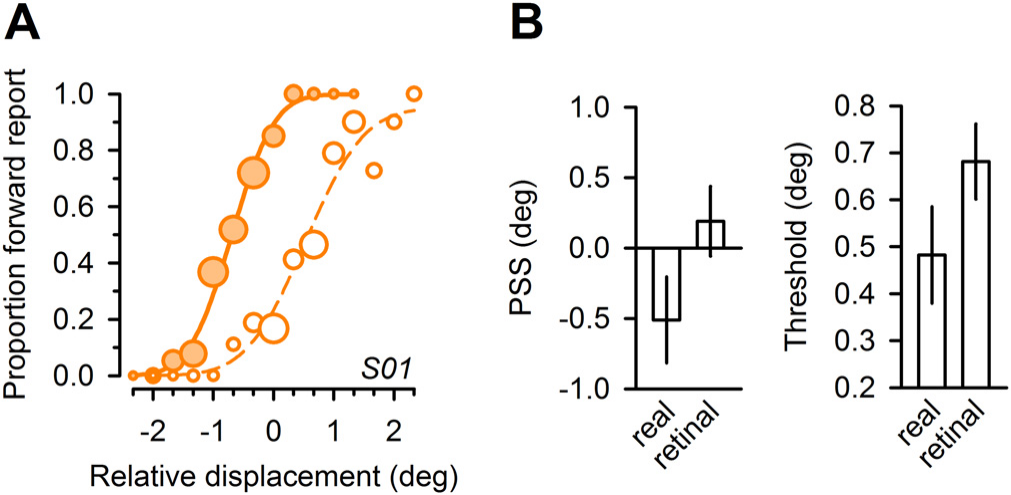
Extraretinal information contributes to perceptual decisions. ***A***, Psychometric functions of naïve subject in the JUMP task (same subject as in Fig. 2; cf., Fig. 2*D*). Proportion of trials in which subject reported an apparent target jump in saccade direction (forward), plotted against relative displacement levels (filled orange circles and continuous line). In addition, data are replotted as a function of the (binned) visual error experienced after saccade completion (unfilled orange circles and dashed line). ***B***, Mean group bias (PSS, left panel) and group threshold estimates (right panel) for psychometric functions fitted to perceptual reports with respect to the real target displacement (“real”) and the (binned) visual error experienced after saccade completion (“retinal”). Error bars reflect standard errors of the mean.

Observers were instructed to perform a simple perceptual decision on the apparent displacement direction of a visual target that briefly disappeared in sync with saccadic eye movement (JUMP condition). In this visually sparse environment, performance is assumed to be driven by internal monitoring of eye movement metrics [12, 32–35]. We estimated accuracy and precision in this task by determining the bias (PSS, point of subjective stationarity) and standard deviation (threshold) of cumulative gaussians, fitted to the individual proportion of apparent ‘forward’ jumps for a given displacement level (see Fig. 2*D* for performance of a representative naïve subject and Fig. 3, dashed orange lines, for group results). We observed a small backward bias for PSS estimates (average PSS, -0.51 deg), corresponding to a systematic tendency to report a forward jump of the target for stationary targets. This bias would be consistent with an internal *over*estimation of actual saccade amplitudes and we note that similar findings were reported in a recent study that also utilized a stimulus overlap design (cf. [40], their Fig. 4*C*). Additional analyses confirmed that subjects used non-visual information for their perceptual decision, over and above the visual error experienced after saccade execution: Fitting psychometric functions to the perceptual data, now binned for the postsaccadic visual error, yielded higher thresholds by a factor of on average 1.41 (Fig. 4). This replicates previous findings [12] that performance in this task rather corresponds to real target jumps, independent from self-induced targeting errors [32, 34, 35].

In a critical test condition (COMBI), the saccade target was presented together with a flanker object (a vertical bar). Perceptual decisions in this condition could also take into account the spatial distance between the target and the flanker (1.2°, 2.8° and 4.4°). Average precision was better than in the JUMP condition for the close and intermediate target-flanker distance and similar to the JUMP condition for the large distance (Fig. 2*F* and Fig. 3*B*, green circles). We assured that changes in perceptual performance were not simply a consequence of altered saccade metrics: Systematic saccade error was 0.61 (± 0.25) deg on average (± S.D.), corresponding to a small saccadic undershoot. This systematic error was slightly larger in JUMP condition [0.83 (± 0.36) deg] compared to COMBI condition [0.53 (±0.16) deg], two-tailed pairwise t-test, *P* = 0.12. No significant differences in systematic errors emerged across different target-flanker distances in the COMBI task [average saccade error (± S.D.) for target-flanker distance of 1.2, 2.8 and 4.4 deg was 0.51 (±0.16), 0.54 (±0.16) and 0.55 (±0.21) deg, respectively], repeated measures ANOVA, *P* = 0.7. Importantly, the same was true for variable saccade errors, as assessed by one standard deviation of individual saccade targeting errors: Average variability (± S.D.) was 0.71 (±0.18) deg in JUMP task versus 0.61 (±0.09), 0.73 (±0.14) and 0.66 (±0.1) deg for target-flanker distances of 1.2, 2.8 and 4.4 deg, respectively (pairwise t-test for JUMP versus COMBI, *P* = 0.39; repeated measures ANOVA across different distances in COMBI, *P* = 0.14).

Improved performance might arise from a strategy switch, with perceptual decisions now based on visual relational information. Alternatively, subjects might take into account both pieces of information and integrate them into a combined estimate of target displacement. We therefore created a control condition (VISUAL), in which we asked subjects to estimate the relative flanker displacement with respect to the target dot without intervening eye movements. This condition mimicked the test condition (COMBI) in terms of afferent input, but subjects were instructed to keep fixating, while the target-flanker pair was first presented in the periphery and then moved to the subject’s fixation point.

Precision of displacement estimates in this condition decreased with larger distances (Fig. 2*E* and Fig. 3*B*, blue circles). Critically, perceptual precision in this condition was much worse for the intermediate and large distance compared to the COMBI condition, and indistinguishable in both conditions for the close distance. This suggests that subjects indeed used a combination of both pieces of information in the COMBI condition, as also supported by intermediate perceptual biases in this condition as compared to the two single cue conditions (Fig. 3*A*, green circles).

**Figure 5:**
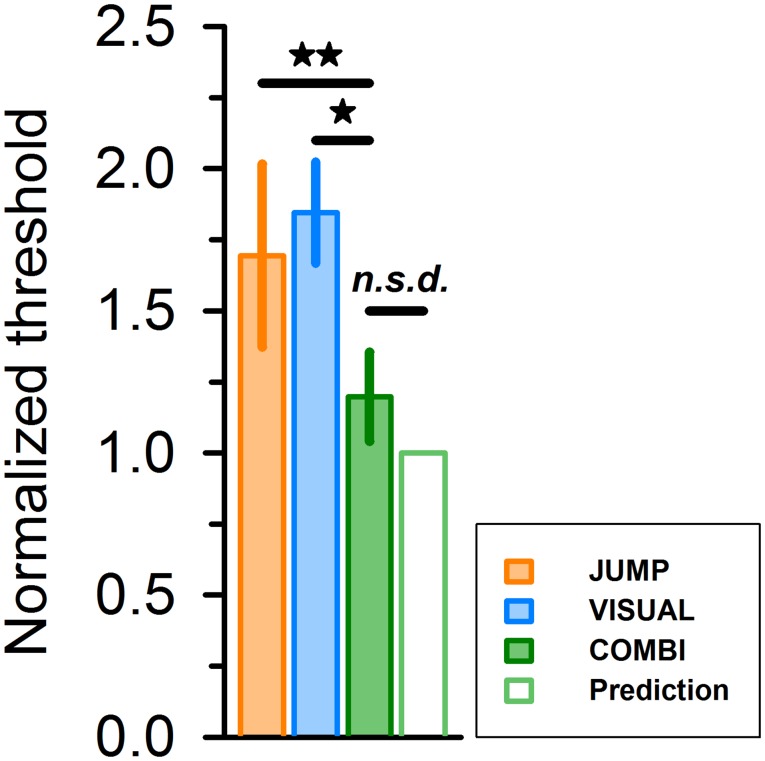
Average group thresholds for different conditions, after normalizing to unity for the predicted value (unfilled green bar to right). Error bars represent standard error of the mean. Statistical comparison yielded significant differences for COMBI vs. JUMP and COMBI vs. VISUAL, but no significant difference for COMBI vs. predicted thresholds (two-tailed pairwise t-test on non-normalized data, * *P* < 0.05; ** *P* = 0.01).

To simulate performance in the combined condition as integration of extraretinal and afferent relational cues, we used predictions from a simple cue integration model [see Materials and Methods, eq. (3)]. Drawing on the average bias and precision estimates in the JUMP and VISUAL conditions, the model predicted parameters for the three target-flanker distances in the COMBI condition that were close to the empirically observed averages in our sample of subjects (Fig. 3*B*, green diamonds). For comparison of thresholds across conditions, we normalized individual thresholds to unity for the predicted value [30]. Thresholds improved in COMBI condition (Fig. 5, filled green bar) over a grand average of single cue conditions (JUMP, orange bar; VISUAL, blue bar) by a factor of 1.66, compared with a predicted value of 1.77. Statistical analysis indicated a significant difference between single cue thresholds and the thresholds observed in the COMBI condition (non-normalized values, two-tailed paired t-test, *P* ≤ 0.038). By contrast, thresholds in the COMBI condition were statistically indistinguishable from thresholds predicted by optimum integration (*P* =.81).

Perceptual judgments in single-cue conditions exhibited different biases (Fig. 3*A*). We therefore also tested for an additional prediction of optimal cue integration: Biases in COMBI condition should correspond to a weighted average of single cue biases, according to their relative reliability [see Materials and Methods, eqs. (2), (3)]. Comparison of average biases in the COMBI condition suggests a close correspondence to predicted values (Fig. 3*A*, green diamonds). However, differences across conditions were comparatively small and not significantly different from each other (two-tailed paired t-tests, PSS values of single cue conditions versus COMBI condition, all *P’*s ≥ 0.17). We note that our task was designed to demonstrate an improvement of perceptual performance (i.e., reduction of variance) in the COMBI condition versus single cue conditions, consistent with optimal cue integration. A modified design with insertion of experimentally induced cue conflicts (i.e., displacing the flanker within the saccade by small amounts, cf. [31]) may allow for a stronger inference on reliability-based reweighting of single cue information [29–31].

## Discussion

Different mechanisms have been proposed to ensure the seemingly flawless maintenance of perceptual stability across eye movements [41]. First, uniform whole-field translations with preserved relational information within a visual scene could be taken as a strong indicator of external stability [23]. Second, the visuomotor system may resort to eye position information provided by afferent, proprioceptive input [5, 8]. Third, the outflow, efferent oculomotor signal could be used to predict impending visual changes [3, 4, 6, 7]. Previous experimental evidence was largely taken as evidence for a predominant reliance on reafferent relational cues whenever they are available [24, 25]. On the other hand, an important role of outflow CD signals for the matching of visual space was demonstrated in experimental settings where no relational information could be used [1].

Our results suggest that outside the sparse environment of a well-controlled laboratory setting, matching of visual space across eye movements is based upon an obligatory integration of both afferent relational information and internal eye position signals. Our findings are consistent with a close to optimal weighting of both cues according to their respective reliability, similar to the integration of different sensory channels in multisensory integration. A weighted and adaptive integration of both cues seems ecologically desirable, as both noise within the visuomotor system and reliability of visual context information constantly changes in everyday life. For example, reliability of visual cues may range from browsing static scenes like a living room with high-quality, stable relational information [23] to a night-time traffic situation which involves visible landmarks that move in different directions at different speeds within a generally deprived and unstable environment.

A weighted integration of retinal and extraretinal signals could also offer a parsimonious explanation for previous experimental findings, where flanker objects in a similar task were shown to bias the displacement detection for temporarily blanked targets [25]. Critically, the bias was shown to decline with increasing target-flanker distances in a gradual fashion [25]. This would be expected if the visuomotor system considered the (decreasing) reliability of relational information and accordingly weights down its influence compared to internal monitoring information. In our experiment, we aimed to empirically assess reliability of these two single cues to generate predictions on how subjects should combine them for different reliability levels of visual relational information. Our findings provide a mechanistic approach that allows for specific predictions regarding the spatial range over which relational information will impact displacement detection across saccades. As already suggested in a previous study [25], this spatial range should also depend on eye movement metrics, as signal-dependent noise and thus reliability of oculomotor CD will vary with saccade amplitude [13, 42] or number of subsequent saccades in eye movement sequences [2, 10].

We note that the design of the VISUAL single-cue condition may only represent a rough proxy to the relational context information actually transferred across eye movements. First, stimulus dynamics in the VISUAL condition did not reproduce idiosyncratic saccade landing errors. Furthermore, different instructions and a differential distribution of attention resources in the COMBI condition may influence perceptual performance [43]. In this context it remains an open question how our findings might translate to more cluttered visual scenes typically encountered in everyday life. Previous studies indicate that the structural gist of background information is indeed taken into account for matching of visual space across saccades [44, 45]. Reliability of such information will be more difficult to parameterize and the visuomotor system may additionally incorporate a non-flat prior on (the implausibility of) visual background jumps during eye movements [46]. Such a prior may also explain the general phenomenon of saccadic suppression of displacement (SSD), i.e., the failure to detect intrasaccadic jumps of visual targets that are directly available after saccade completion [12, 13, 25].

Visual reafference and oculomotor CD information might not be the only cues to match visual space across eye movements. We note a recent study indicated that for longer sequences of saccades in the dark, proprioceptive eye position information might increasingly be taken into account as an additional afferent cue [10]. Indeed, a relevant contribution of proprioceptive inflow information for the integration of space across eye movements is also suggested by previous work ([8, 9], but see [2]). It is unclear where and how the integration of these different pieces of information may be accomplished within the visuomotor system, but neuronal populations in areas such as the posterior parietal cortex have been shown to represent these signals [47]. Reliability of different cues might in this context be coded implicitly by the spread of population codes without the need for explicit calculations of uncertainty [48].

On a different note, traditional views hold that compared to passive fixation, saccadic eye movements entail additional processing costs and should be accompanied by noisier encoding of spatial relationships. In line with comparable findings in reaching tasks [49], our results on the contrary suggest that active oculomotor exploration might actually aid the estimation of spatial relations by adding internal predictions of visual reafference to a purely retinotopic representation.
